# Complete genome sequence of *Bradyrhizobium lupini* LLZ14, a nitrogen-fixing and plant growth-promoting bacterium

**DOI:** 10.1128/mra.00935-24

**Published:** 2025-01-29

**Authors:** Zohra Chaddad, Omar Bouhnik, Mouad Lamrabet, Soufiane Alami, Mustapha Missbah El Idrissi

**Affiliations:** 1 Centre de Biotechnologies Végétales et Microbiennes, Biodiversité et Environnement, Faculty of Sciences, Mohammed V University in Rabat, Rabat, Morocco; University of Strathclyde, Glasgow, United Kingdom

**Keywords:** *Bradyrhizobium lupini*, *Lupinus luteus*, plant growth promotion, rhizobium-legume symbiosis

## Abstract

In this study, we present the complete genome of *Bradyrhizobium lupini* LLZ14, a nodule-forming bacterium isolated from *Lupinus luteus* root nodules with high plant growth-promoting abilities. This genome contains genes predicted to be involved in plant stress tolerance and growth promotion, including auxin production, phosphatase, and 1-aminocyclopropane-1-carboxylate deaminase.

## ANNOUNCEMENT

Nodule-forming *Bradyrhizobium* spp. can promote plant growth by enhancing nutrient uptake and tolerance to various stresses, in addition to their ability to fix nitrogen in symbiosis with legumes (1). In this work, we sequenced the genome of *Bradyrhizobium* sp. LLZ14, a strain selected for its high growth-promoting ability in *Lupinus luteus* under nitrogen-free conditions (2). A total of 152 nodules were collected from 18 lupin plants, obtained after seed disinfection and greenhouse cultivation in soils from agricultural lands of Morocco (33°48′50.8″N, 6°30′07.5″W). The nodules were disinfected using 2.5% NaClO for 3 min, crushed, and streaked on yeast extract mannitol medium (3) at 28°C. The isolates were purified by repeated single colony streaking and tested for their ability to improve plant growth (2).

DNA was extracted from the liquid culture in TY medium (4) (28°C, 150 rpm) using the PureLink Genomic DNA MiniKit (Invitrogen) and quantified with a NanoDrop ND2000/2000c (ThermoFisher Scientific). The library was prepared using the Rapid Barcoding Sequencing Kit (SQK-RBK004) and then sequenced on a FLO-MIN106D flow-cell for 72 hours on a GridION X5 (Oxford Nanopore Technologies, UK), with filtering reads shorter than 200 bases. Basecalling was performed with the fast module in Dorado software (version 7.3.11) included in the MinKNOW app (version 24.02.16).

The obtained reads were processed prior to *de novo* assembly using LongQC (version 1.2.1) for quality check (5), Porechop (version 0.2.4) to trim adapters (6), and nanofilt (version 2.8.0) to filter low-quality sequences (7). The *de novo* assembling was performed using Canu (version 2.3) (8), resulting in a single contig that was polished using Racon (version 1.5.0) (9) and Medaka (version 1.11.3) (10). The output was checked using BUSCO (version 5.7.1) with alphaproteobacteria_odb10 data set (11) and Quast (version 5.2.0) (12). The NCBI Prokaryotic Genome Annotation Pipeline (version 6.7) (13) was used to perform the annotation. Default parameters were used for all software. The summary results of the sequencing, assembly, and annotation are presented in Table 1. The LLZ14 strain genome is predicted to contain genes involved in nodulation as nodulation factors and others involved in plant growth-promoting activities, including 1-aminocyclopropane-1-carboxylate deaminase, phosphate solubilization, and auxin production genes.

**TABLE 1:** Sequencing, *de novo* assembly, and annotation summary results of the sequenced genome

Parameters	*Bradyrhizobium lupini* LLZ14
Number of raw reads	33,302
Number of reads^*a*^	27,308
Mean read length (bp)^*a*^	7,822.42
Read *N*_50_ (bp)^*a*^	13,326
Coverage^*a*^	27.29
No. of contigs	1
Assembly size	7,826,318
BUSCO (version 5.7.1) estimated completeness (%)	94.7
GC content (%)	63.37
No. CDSs with protein^*b*^	6,828
No. of rRNAs	3
No. of tRNAs	47
No. of ncRNAs	4

^*a*^ After processing of reads.

^*b*^ CDSs, coding DNA sequences.

The digital DNA-DNA hybridization for the LLZ14 genome was calculated using GGDC 3.0 (https://ggdc.dsmz.de/ggdc.php) (14), revealing a highest value of 57.1% with *Bradyrhizobium canariense* BTA-1^T^ (GCF_019402665.1) using formula 2. These results were verified by analyzing the average nucleotide identity using Pyani (version 0.2.12) (15) and showed values below 93.9% as compared to the available closest type strain genomes.

**Fig 1:**
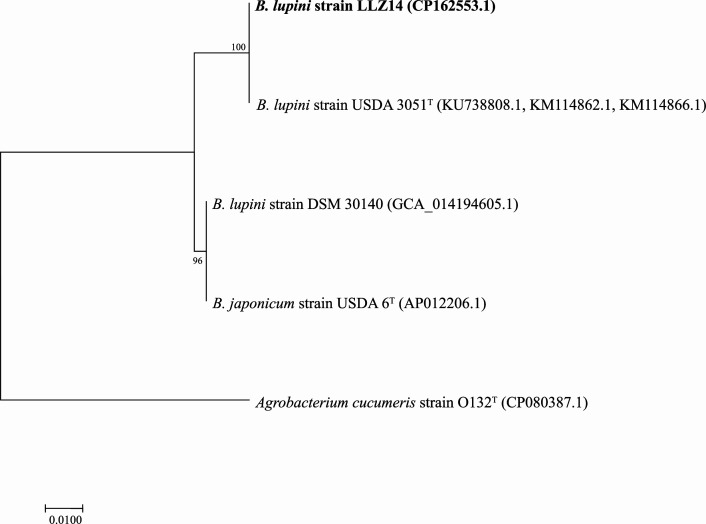
Maximum likelihood phylogenetic tree of concatenated nucleotide sequences from *atpD*, *glnII*, and *recA* genes (2,382 bp) comparing the LLZ14 strain with *Bradyrhizobium japonicum* and *B. lupini* type strains, and *Bradyrhizobium lupini* DSM30140 in the NCBI database. The results revealed that strain LLZ14 is closely related to *B. lupini* USDA3051^T^, while strain DSM30140 is rather closer to *B. japonicum* USDA6^T^. Nucleotide sequence alignments were performed using the CLUSTAL W algorithm. The phylogenetic tree was inferred using the maximum likelihood method with the Hasegawa-Kishino-Yano substitution model, codon position set to 1. Bootstrap percentages, based on 1,000 replicates, are shown next to each node. The nucleotide substitution frequency is indicated by the scale bar below the tree. The accession numbers of the sequences are indicated inside parenthesis, and the subscript T indicates the type strains. The tree was outrooted using *Agrobacterium cucumeris* strains O132^T^.

The concatenation of four gene markers in our previous publication showed over 99% nucleotide sequence identity with the type strain *Bradyrhizobium lupini* USDA3051^T^ (3). However, the genome of this type strain is unavailable in any database with only a few gene markers in the NCBI database. The maximum-likelihood phylogenetic analysis using MEGA11 (16) of concatenated *atpD*, *glnII*, and *recA* sequences (2,382 bp) from our genome, type strains, revealed that LLZ14 is closely related to USDA3051^T^ (Fig. 1).

## Data Availability

The LLZ14 genome is available in GenBank CP162553.1 under NCBI BioProject PRJNA1137243 and BioSample SAMN42574413. The raw sequencing reads are available in the Sequence Read Archive (SRA) SRX25490475.
